# Design and Position Control of a Bionic Joint Actuated by Shape Memory Alloy Wires

**DOI:** 10.3390/biomimetics9040210

**Published:** 2024-03-30

**Authors:** Yida Zhu, Zhikun Jia, Xiaojie Niu, Erbao Dong

**Affiliations:** 1 CAS Key Laboratory of Mechanical Behavior and Design of Materials, Department of Precision Machinery and Precision Instrumentation, University of Science and Technology of China, Hefei 230026, China; zydyida@mail.ustc.edu.cn (Y.Z.); jzk1997@mail.ustc.edu.cn (Z.J.); 2 Institute of Advanced Technology, University of Science and Technology of China, Hefei 230026, China; mr12@mail.ustc.edu.cn

**Keywords:** bionic joint, shape memory alloy, differential pulley set, position control

## Abstract

Bionic joints are crucial for robotic motion and are a hot topic in robotics research. Among various actuators for joints, shape memory alloys (SMAs) have attracted significant interest due to their similarity to natural muscles. SMA exhibits the shape memory effect (SME) based on martensite-to-austenite transformation and its inverse, which allows for force and displacement output through low-voltage heating. However, one of the main challenges with SMA is its limited axial stroke. In this article, a bionic joint based on SMA wires and a differential pulley set structure was proposed. The axial stroke of the SMA wires was converted into rotational motion by the stroke amplification of the differential pulley set, enabling the joint to rotate by a sufficient angle. We modeled the bionic joint and designed a proportional–integral (PI) controller. We demonstrated that the bionic joint exhibited good position control performance, achieving a rotation angle range of −30° to 30°. The proposed bionic joint, utilizing SMA wires and a differential pulley set, offers an innovative solution for enhancing the range of motion in SMA actuated bionic joints.

## 1. Introduction

Bionic joints, a crucial component in robotics, are currently the focus of extensive research. Their primary objective is to mimic the rotational motion and flexibility inherent in human and animal joints through the integration of mechanical structures, actuators, transmission methods, sensors, and controllers. Bionic joints can be categorized into several groups based on their actuators: electric motors [1,2], hydraulic actuators [3,4,5], pneumatic actuators [6,7,8], and smart material actuators (such as piezoelectric actuators [9,10,11,12], electroactive polymer actuators [13,14,15], shape memory alloy (SMA) actuators [16,17,18,19,20], and twisted and coiled polymer actuators [20,21]).

Compared to traditional actuators like electric motors, hydraulic actuators, and pneumatic actuators, SMAs have the advantages of high energy density, substantial load capacity, light weight, quiet operation, and low actuating voltage [22]. SMAs exhibit two distinct phases: high-temperature austenite and low-temperature martensite, depending on mechanical stress and temperature variations [23]. SMAs have two thermo-mechanical properties: shape memory effect (SME) and pseudo-elasticity (PE) [24]. The SME of SMAs is characterized by the ability to fully recover their original shape and volume after experiencing significant deformation. This recovery occurs when the material is heated above a certain temperature threshold, typically the martensite start temperature, which triggers the martensite-to-austenite phase transformation. In the PE of SMAs, applying external force to the SMA at a temperature slightly higher than the martensite start temperature triggers the austenite-to-martensite phase transformation, which leads to plastic deformation. Once the external force is removed, the SMA returns to its original shape and the strain disappears.

**Figure 1:**
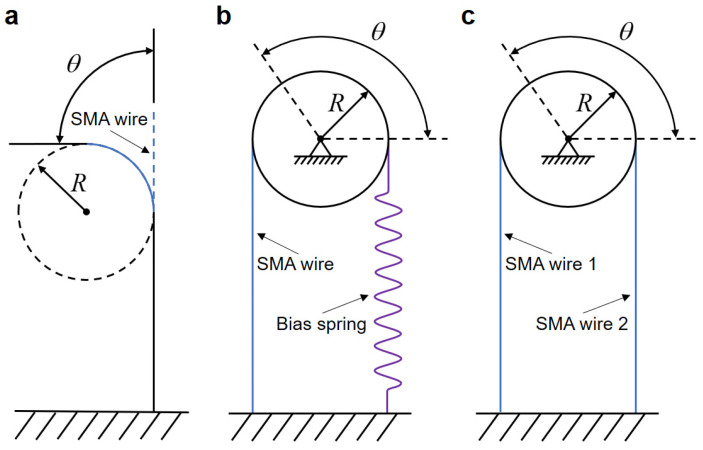
Types of SMA wire-actuated bionic joints. (**a**) Single SMA wire actuated. (**b**) Biased SMA wire actuated. (**c**) Differential SMA wire actuated.

SMA wires can be used as actuators for bionic joints to achieve bi-directional rotation. As shown in Figure 1, SMA wire-actuated bionic joints are broadly categorized into three types: single SMA wire-actuated joints, biased SMA wire-actuated joints, and differential SMA wire-actuated joints [25]. Single SMA wire-actuated joints utilize thick two-way SMA wires exhibiting the two-way SME to achieve bi-directional rotation of the bionic joints. This means that the SMA wires are capable of remembering their shape at both high and low temperatures. Bias SMA wire-actuated joints can have an active SMA wire on one side and a bias spring on the other side, which is slow to respond as the speed is determined by the cooling process and the bias spring. A differential SMA wire-actuated joint consists of two antagonistic SMA wires and responds faster than a biased SMA wire-actuated joint, but it consumes more power, and the angle is limited by the rigid antagonistic SMA wires. The rotational angle of the SMA wire-actuated bionic joint is related to the effective output stroke of SMA wires. The maximum recoverable strain for repeatable motion of commonly used two-way SMA wires ranges from 2% to 5% [26]. In order to achieve the wide range of motion in SMA wire-actuated joints, researchers have made some attempts. Baek et al. [27] employed a single two-way SMA wire in a double-bend configuration as an actuator for the joint, enabling bi-directional and large-range angular output. Takumi and Sumito [28] used SMA wires in combination with bias springs to actuate a micro-hand to realize a wide range of hand flexion and extension. Guo et al. [29] designed a compliant differential SMA rotary joint, which was combined with a normal torsion spring and actuated by two SMA wires, to achieve a wide range of rotary joint motion. Britz et al. [30] introduced a modular SMA wire rotary actuator, achieving a large rotational range through accumulated small motions from individual modules, enabled by an agonist/antagonist SMA wire configuration, and offering scalability for custom rotation and torque. Jia et al. [19] implemented a series of multiple smart digital structures, constructed with SMA wires, to form an SMA artificial muscle matrix for multilevel angular control of the bionic mechanical ankle. Another way to increase the range of joint motion is by utilizing long SMA wires wrapped around multiple fixed pulleys within a limited space [31].

This article introduces a solution to the limited axial stroke of SMA wires by proposing a bionic joint that incorporates SMA wires and a differential pulley set. The novel design of this differential pulley set structure comprises a double-diameter fixed pulley and two movable pulleys. By leveraging the radius difference of the double-diameter fixed pulley, this design amplifies the axial stroke of 80 mm long SMA wires, resulting in a bionic joint that can achieve an output angle ranging from −30° to 30°.

The remainder of this article is structured as follows: Section 2 describes the design of the bionic joint. Section 3 presents the mathematical modeling of the SMA wire and the bionic joint, along with the design of a proportional–integral (PI) controller. Section 4 is dedicated to the results of open-loop simulation and experimental testing, as well as closed-loop experiments, including position step response and position tracking response experiments. It also compares the performance of our bionic joint with other differential SMA wire-actuated joints. Finally, this article is concluded in Section 5.

## 2. Novel Bionic Joint Design

**Figure 2:**
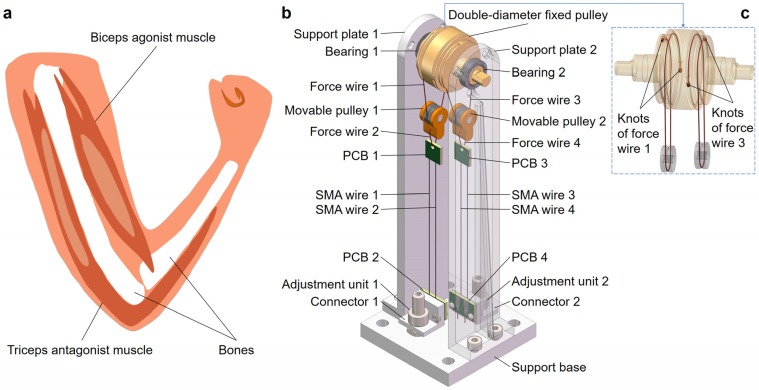
The structural design of the bionic joint. (**a**) Structure of the human elbow joint. (**b**) Three-dimensional isometric view of the bionic joint (support plate 2 is provided with transparency). (**c**) Routing and anti-slip fixation methods for force wires 1 and 3.

The flexion and extension activities of human joints require the synergistic interaction of agonist and antagonist muscles. As illustrated in Figure 2a, during elbow flexion, the biceps muscle acts as an agonist, contracting to facilitate movement, while the opposing triceps muscle remains relaxed and extended. In contrast, during elbow extension, the roles of the biceps and triceps are reversed. This synchronized action between the muscles ensures a smooth execution of joint movements. In our bionic joint design, we emulated this natural mechanism by strategically positioning SMA wires on either side of the joint. The left SMA wires were responsible for facilitating flexion, while the right ones facilitated extension, replicating the precise functionality of the natural joint.

Figure 2b depicts a 3D isometric view of the bionic joint. The external dimensions of the bionic joint are shown in Table 1.

The core structure of the bionic joint was a differential pulley set designed for stroke amplification. The differential pulley set consisted of a double-diameter fixed pulley and two movable pulleys. The double-diameter fixed pulley had two centrosymmetric wire grooves at the middle ends, with dimensions detailed in Table 1. The double-diameter fixed pulley, with bearings (model of both: 6700ZZ, inner diameter: 10 mm, outer diameter: 15 mm, thickness: 4 mm, manufactured by Nedel XUDZ Inc., Yokohama, Japan) on both sides, was connected to the support plates. Movable pulleys 1 and 2 each comprised two connecting housings and a V-groove bearing (model: 623-V, inner diameter: 3 mm, outer diameter: 10 mm, thickness: 4 mm, width of groove: 1.2 mm, depth of groove: 0.8 mm, manufactured by ZZFY Inc., Harbin, China). As depicted in Figure 2c, movable pulley 1 was seamlessly connected to the double-diameter fixed pulley through a meticulous routing and fixing process. One end of force wire 1 was threaded through the large-diameter groove hole of the double-diameter fixed pulley and securely knotting it. Force wire 1 then followed the large-diameter groove of the fixed pulley, routing it through the lower side of the V-groove bearing, and subsequently wrapping around the small-diameter groove of the fixed pulley. The other end of force wire 1 was then threaded through the small-diameter groove hole of the double-diameter fixed pulley and knotted securely. The knots of force wire 1 were strengthened by threading the wire through the groove hole and securely tying it with three consecutively overlapping overhand knots. This ensures a sturdy connection, effectively preventing force wire 1 from slipping off the double-diameter fixed pulley. The lower end of movable pulley 1 was connected to the winding of the printed circuit board (PCB) 1 via the force wire 2. The movable pulley 2 and force wires 3 and 4 were connected in a similar manner to movable pulley 1 and force wires 1 and 2, as described above.

The SMA wires were made of a nickel–titanium alloy (90 °C Flexinol actuator wires, manufactured by DYNALLOY, Inc., Irvine, CA, USA). SMA wires were arranged in parallel between the PCBs. The dimensions of SMA wires and PCBs are in Table 1. Notably, PCBs 1 and 3 both featured a hole with a diameter of 2 mm for the connection of the force wire, as well as two holes with a diameter of 0.33 mm for the secure fixation of SMA wires. Conversely, PCBs 2 and 4 were both equipped with symmetrical solder pads, each containing two holes with a diameter of 0.91 mm to facilitate the winding and soldering of SMA wires. Additionally, there were two holes with a diameter of 0.91 mm designated for soldering enameled round copper wires, with the ends of the wires stripped of approximately 8 mm of insulation. To ensure a secure attachment, the solder mask area of PCB 2 was equipped with two symmetrical holes positioned away from the solder pads, each with a diameter of 2 mm.

PCBs 2 and 4 were fixed with connectors 1 and 2, respectively, and they were attached to the support base by the adjustment units, which both consisted of a bolt and a round nut. The round nut could be rotated up and down to adjust the tightness of the SMA wires. The support base and support plates were fixed together by means of hexagonal bolts and nuts.

**Figure 3:**
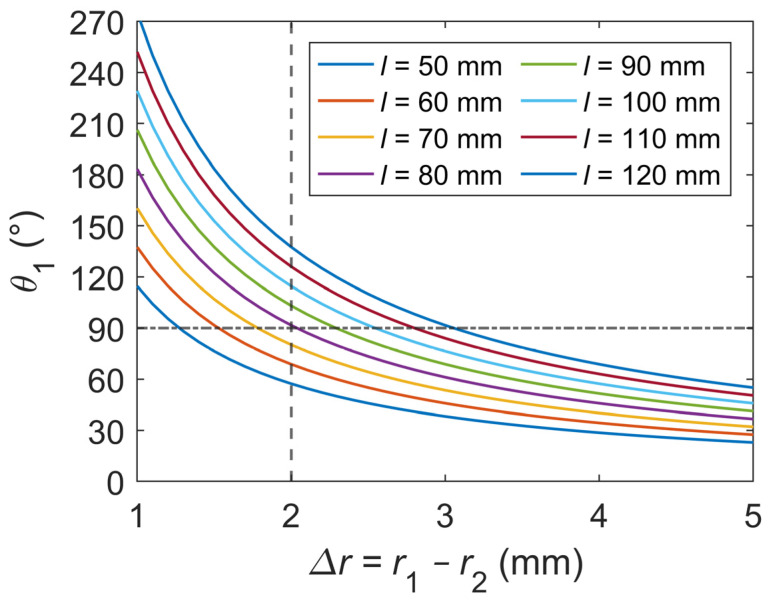
Parametric analysis for the bionic joint design. l is the length of the SMA wire. $r_{1}$ and $r_{2}$ represent the radii of the large groove and small groove on the double-diameter fixed pulley, respectively. $\theta_{1}$ represents the rotation angle of the double-diameter fixed pulley.

The parametric analysis for the bionic joint design, as shown in Figure 3, relies on Equation (12), whose derivation is comprehensively outlined in Section 3. To analyze the length of each SMA wire utilized in the bionic joint, discrete intervals of 10 mm were established for the length of the SMA wire, ranging from 50 to 120 mm. Additionally, a continuous range was considered for the difference in radii of the double-diameter fixed pulley, varying from 1 to 5 mm. The maximum strain value for the SMA wire was set to 0.02, and the expected angle without differential settings for a single-sided SMA wire was targeted to reach 90°, while the actual differential setting was approximately 30°. Based on Figure 3, it was determined that a length of 80 mm for the SMA wire is suitable when the difference in radii of the double-diameter fixed pulley is 2 mm.

## 3. Bionic Joint Modeling and Controller Design

### 3.1. SMA Wire Modeling

#### 3.1.1. Constitutive Model

Based on previous research [32,33], the constitutive model of the SMA wire can be described by the relationship between the strain rate, temperature rate, and martensite volume fraction rate. The constitutive model of the SMA wire is usually in differential form and can be expressed as

(1) $$\dot{\sigma }=E\dot{\varepsilon }+\Theta \dot{T}+\Omega \dot{\xi }$$

where σ, ε, T, and ξ, correspondingly, are defined as the stress, strain, temperature, and martensite volume fraction of the SMA wire. Also, E is the modulus of elasticity (Young’s modulus), Θ is the thermoelastic coefficient, and Ω is the phase transformation coefficient. The value of Θ is usually small and can often be neglected when the temperature rate is not large.

Young’s modulus of the SMA wire is usually approximated as being linearly related to the proportion of the composition of the individual phases; hence, the expression for the Young’s modulus of the SMA wire is given by

(2) $$E=\left(1-\xi \right)E_{A}+\xi E_{M}$$

where $E_{A}$ and $E_{M}$ are austenite Young’s modulus and martensite Young’s modulus of the SMA wire, respectively.

By substituting the initial state (σ=0, ε=0, ξ=0) and the final state (σ=0, $\varepsilon =\varepsilon_{L}$, ξ=1) of the SMA wire into Equation (1) while neglecting the thermoelastic term, we obtain

(3) $$\Omega =-\varepsilon_{L}E$$

where $\varepsilon_{L}$ is maximum recoverable strain of the SMA wire.

According to Equations (1) and (3), the constitutive model of the SMA wire can be simplified as

(4) $$\dot{\sigma }=E\left(\dot{\varepsilon }-\varepsilon_{L}\dot{\xi }\right).$$

Considering the initial state ($\sigma_{0}$, $\varepsilon_{0}$, $\xi_{0}$) of the SMA wire, the stress of the SMA wire is

(5) $$\sigma =\sigma_{0}+\int_{0}^{t}E\left(\dot{\varepsilon }-\varepsilon_{L}\dot{\xi }\right)dt.$$

The contraction force of the SMA wire is

(6) F=Aσ

where A is the cross-sectional area of the SMA wire, $A=\pi d^{2}/4$, and d is the diameter of the SMA wire.

#### 3.1.2. Phase Transformation Model

The Liang–Rogers model [33] assumes that the martensite volume fraction is related to the cosine function of temperature and stress and proposes a cosine function-type phase transformation model.

In the SMA cooling process, the martensite volume fraction ξ of the austenite-to-martensite phase transformation is given by

(7) $$\begin{array}{cc} \xi =\frac{1-\xi_{A}}{2}\cos\left[a_{M}\left(T-M_{f}\right)+b_{M}\sigma \right]+\frac{1+\xi_{A}}{2} & \mathrm{if}\;M_{f}+\frac{\sigma }{C_{M}}\leq T\leq M_{s}+\frac{\sigma }{C_{M}} \end{array}$$

In the SMA heating process, the martensite volume fraction ξ of the martensite-to-austenite phase transformation is given by

(8) $$\begin{array}{cc} \xi =\frac{\xi_{M}}{2}\cos\left[a_{A}\left(T-A_{s}\right)+b_{A}\sigma \right]+\frac{\xi_{M}}{2} & \mathrm{if}\;A_{s}+\frac{\sigma }{C_{A}}\leq T\leq A_{f}+\frac{\sigma }{C_{A}} \end{array}$$

where $a_{M}=\frac{\pi }{M_{s}-M_{f}}$, $a_{A}=\frac{\pi }{A_{f}-A_{s}}$, $b_{M}=-\frac{a_{M}}{C_{M}}$, and $b_{A}=-\frac{a_{A}}{C_{A}}$. $A_{s}$, $A_{f}$, $M_{s}$, and $M_{f}$ represent the start and finish temperatures of the austenite-to-martensite and martensite-to-austenite phase transformations of the SMA wire, respectively. $C_{A}$ and $C_{M}$ represent the stress influence coefficients of the austenite and martensite phases, respectively. $\xi_{A}$ and $\xi_{M}$ are the initial martensite volume fractions of the austenite-to-martensite and martensite-to-austenite phase transformations, respectively.

#### 3.1.3. Heat Transfer Model

In our experimental setup, the heat required for the martensite-to-austenite phase transformation of the SMA wire was generated by the Joule heating effect resulting from the voltage applied to the SMA wire. The heat transfer model outlines the relationship between the temperature rate of the SMA wire and the Joule heat generated by energizing the wire, along with the heat energy lost due to ambient convective heat transfer. The heat transfer equation for the SMA wire is given by

(9) $$mc_{p}\dot{T}=V^{2}/R-Sh\left(T-T_{0}\right)$$

where m is the mass of the SMA wire, m=Alρ; l is the length of the SMA wire; ρ is the density of the SMA wire; $c_{p}$ is the specific heat capacity of the SMA wire; V is the voltage applied to the SMA wire; R is the resistance of the SMA wire, $R=R_{0}l$; $R_{0}$ is the resistance per unit length of the SMA wire; S is the surface area of the SMA wire, S=πdl; h is the heat convective coefficient, which is a second-order polynomial in temperature with parameters $h_{0}$ and $h_{2}$, $h=h_{0}+h_{2}T^{2}$; and $T_{0}$ is the ambient temperature.

### 3.2. Bionic Joint Modeling

**Figure 4:**
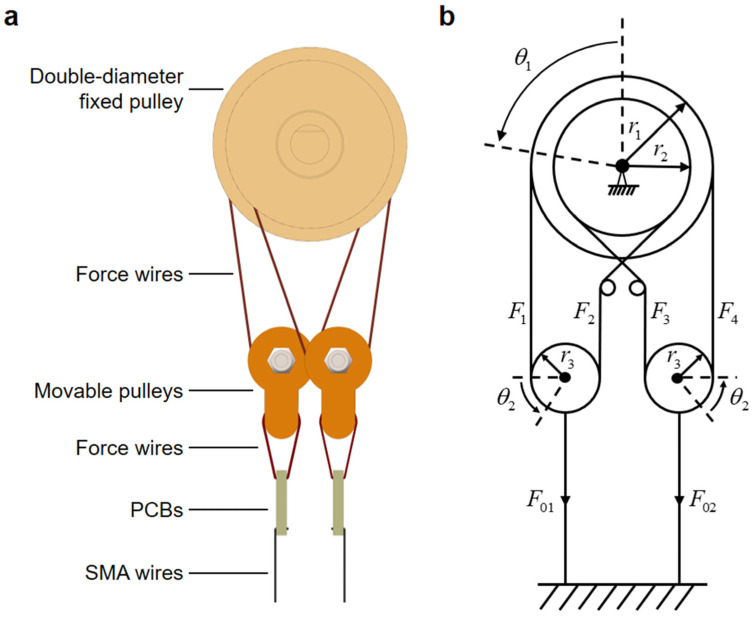
Bionic joint modeling. (**a**) Focused side view of the bionic joint’s core structure. (**b**) Schematic diagram of the bionic joint.

Figure 4 showcases the modeling of the bionic joint, with Figure 4a presenting a focused side view of its intricate core structure and Figure 4b offering a schematic diagram of the joint. To simplify the calculation, the force wires at the two ends of the movable pulleys were assumed to be vertical. The contraction force $F_{01}$ generated by the left SMA wires was greater than the contraction force $F_{02}$ generated by the right SMA wires. Since the SMA wires operated in opposite directions, when one side of the SMA wires contracted, the ones on the other side were stretched. The contraction displacement of the left SMA wires and the stretched displacement of the right SMA wires are both denoted as x. The displacements of movable pulleys 1 and 2 are also x. The tensions generated by the force wires at the ends of the movable pulleys are $F_{1}$, $F_{2}$, $F_{3}$, and $F_{4}$, respectively. The rotation angle of the double-diameter fixed pulley is $\theta_{1}$, the radius of the large groove is $r_{1}$, and the radius of the small groove is $r_{2}$. The rotation angles of the groove bearings of movable pulleys 1 and 2 are both $\theta_{2}$. The radius of V-groove bearings is $r_{3}$.

For the bionic joint kinematics model, the displacement x is given by

(10) $$x=l(\varepsilon_{L}-\varepsilon_{1})=l(\varepsilon_{2}-\varepsilon_{L})$$

where $\varepsilon_{1}$ and $\varepsilon_{2}$ are the strains of the left and right SMA wires, respectively. The relationship between the rotation angle $\theta_{1}$ of the double-diameter fixed pulley and the displacement x is given by

(11) $$\theta_{1}=\frac{2}{r_{1}-r_{2}}x$$

where $\frac{2}{r_{1}-r_{2}}$ is the transmission ratio, which determines the stroke amplification effect. Combining Equations (10) and (11), the relationship between $\theta_{1}$, $\varepsilon_{1}$, and $\varepsilon_{2}$ can be determined as follows:

(12) $$\varepsilon_{L}-\varepsilon_{1}=\varepsilon_{2}-\varepsilon_{L}=\frac{r_{1}-r_{2}}{2l}\theta_{1}.$$

After deriving Equation (12) with respect to time, the strain rates ${\dot{\varepsilon }}_{1}$ and ${\dot{\varepsilon }}_{2}$ of the left and right SMA wires can be expressed as functions of the angular velocity ${\dot{\theta }}_{1}$ of the bionic joint, as follows:

(13) $$-{\dot{\varepsilon }}_{1}={\dot{\varepsilon }}_{2}=\frac{r_{1}-r_{2}}{2l}{\dot{\theta }}_{1}.$$

For the bionic joint dynamics model, considering a double-diameter fixed pulley and two movable pulleys individually, it can be shown that

(14) $$\{\begin{array}{l} J_{1}{\ddot{\theta }}_{1}=F_{1}r_{1}-F_{2}r_{2}+F_{3}r_{2}-F_{4}r_{1}\\ J_{2}{\ddot{\theta }}_{2}=F_{1}r_{3}-F_{2}r_{3}\\ m_{1}\ddot{x}=F_{01}-b_{1}\dot{x}+m_{1}g-F_{1}-F_{2}\\ J_{2}{\ddot{\theta }}_{2}=F_{4}r_{3}-F_{3}r_{3}\\ m_{1}\ddot{x}=F_{3}+F_{4}-F_{02}-b_{2}\dot{x}-m_{1}g \end{array}$$

where $J_{1}$ is the moment of inertia of the double-diameter fixed pulley, $J_{2}$ is the moment of inertia of the V-groove bearing in the movable pulley, $m_{1}$ is the mass of the movable pulley, and g is the acceleration of gravity. $b_{1}$ and $b_{2}$ are the damping coefficients of the left and right SMA wires. By combining Equations (6), (11), and (14) and eliminating the intermediate variables, the angular acceleration ${\ddot{\theta }}_{1}$ of the double-diameter fixed pulley can be expressed as

(15) $${\ddot{\theta }}_{1}=\left[\frac{\left(r_{1}-r_{2}\right)nA}{2}\left(\sigma_{01}-\sigma_{02}\right)-\frac{{\left(r_{1}-r_{2}\right)}^{2}}{4}\left(b_{1}+b_{2}\right){\dot{\theta }}_{1}\right]/\left[J_{1}+\frac{{\left(r_{1}-r_{2}\right)}^{2}m_{1}}{2}\right]$$

where n is the number of parallel SMA wires on each side; $\sigma_{01}$ and $\sigma_{02}$ are the stresses of the left and right SMA wires, respectively.

**Figure 5:**
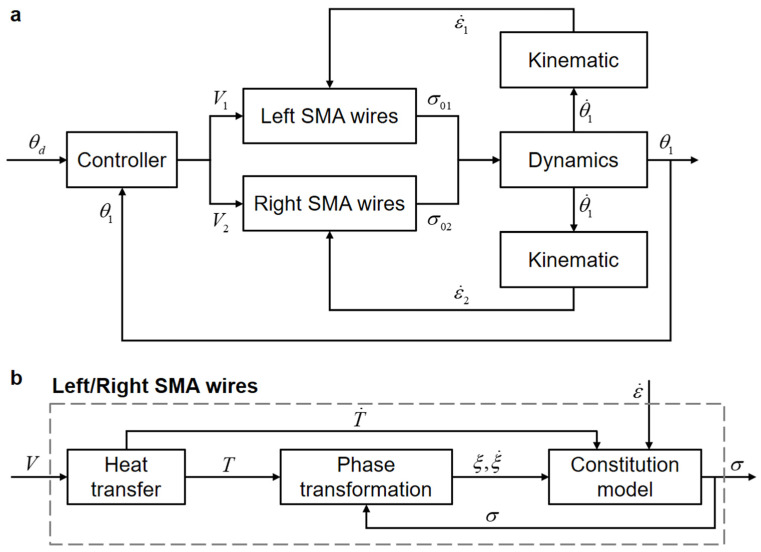
Block diagram of the bionic joint. (**a**) Overall block diagram. (**b**) Sub-block diagram illustrating left/right SMA wires.

Based on the modeling process described above, the block diagrams of the bionic joint model and the SMA wire model are presented in Figure 5a and Figure 5b, respectively. The block diagram of the complete bionic joint model clearly depicts a dual-input single-output (DISO) system exhibiting cross-coupling effects. The controller adjusts the input voltages of the left and right SMA wires, respectively, to ensure that the output angle $\theta_{1}$ tracks the desired angle $\theta_{d}$.

### 3.3. PI Controller Design for Position Control

The position of the bionic joint can be controlled by adjusting the voltages applied to the SMA wires. In this subsection, a PI controller was designed for closed-loop position control of the bionic joint.

**Figure 6:**
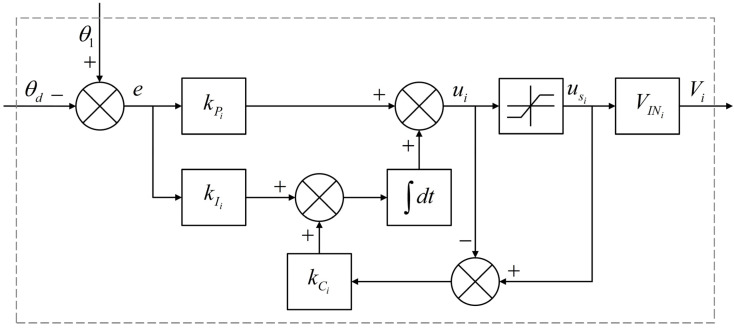
Block diagram of the PI controller with integrator anti-windup.

Figure 6 depicts the block diagram of the PI controller with integrator anti-windup. The angular position error e is defined as

(16) $$e=\theta_{1}-\theta_{d}.$$

The basic PI control law $u_{i}$ is given by

(17) $$u_{i}=k_{P_{i}}e+\left[k_{I_{i}}+k_{C_{i}}(u_{s_{i}}-u_{i})\right]\int edt$$

where $k_{P_{i}}$ and $k_{I_{i}}$ represent the proportional and integral gains, respectively. Integral windup refers to a phenomenon where, due to the integral effect, the output of the PI controller continues to increase in the presence of a system deviation, causing the actuator to reach its limit position. Even if the output continues to rise, the actuator remains inactive, leading to a loss of system control and degraded performance. The anti-windup circuit operates properly to mitigate this issue in the feedback system, even though the limiting value of the saturation function is a time-varying signal [34]. Thus, $u_{s_{i}}-u_{i}$ is employed as the feedback value for the integral part and $k_{C_{i}}$ is the back-calculated gain, $k_{C_{i}}=k_{I_{i}}/k_{P_{i}}$.

To prevent overheating of the SMA wire and the occurrence of negative voltages, the saturation function was utilized. The duty cycle of each metal-oxide-semiconductor field-effect transistor (MOSFET) $u_{s_{i}}$ is calculated as follows:

(18) $$u_{s_{i}}=\{\begin{array}{ll} 1 & \mathrm{if}\;u_{i}>1\\ u_{i} & \mathrm{if}\;0\leq u_{i}\leq 1\\ 0 & \mathrm{if}\;u_{i}<0 \end{array}.$$

The input voltage for each side SMA wires $V_{i}$ is given by

(19) $$V_{i}=V_{IN_{i}}u_{s_{i}}$$

where $V_{IN_{i}}$ represents the input voltage of each MOSFET.

## 4. Experimental Results

### 4.1. Experimental Setup

**Figure 7:**
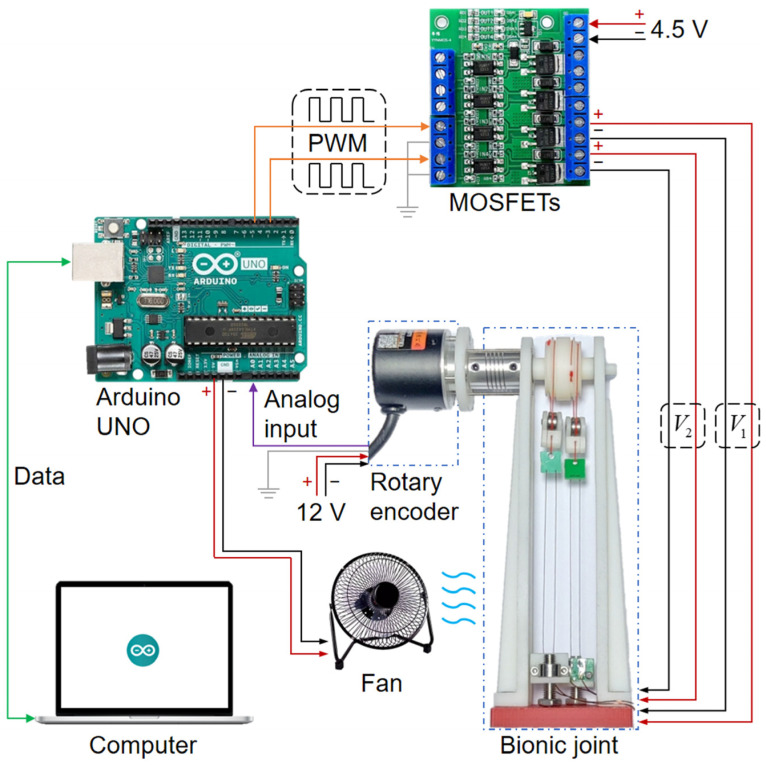
Block diagram of the experimental setup (solid lines in green, purple, and orange represent the signal flows for serial communication, analog input, and PWM output, respectively; solid lines in red, black, and gray represent signals for voltage positive, voltage negative, and signal ground).

The experimental setup was designed to assess the performance of the bionic joint. Figure 7 illustrates the block diagram of the experimental setup. A computer communicated with the Arduino UNO via a serial port for data collection. The rotary encoder (model: BRT38-5V5M1024-RT1, specifications: outer diameter of 38 mm, shaft diameter of 6 mm, analog output range of 0–5 V, precision of 10 bits, manufactured by Briter Electronic Technology Co., Shenzhen, China) recorded the angle of the bionic joint, outputting a 0–5 V analog signal to the Arduino UNO. The rotary encoder was powered by a 12 V supply. The Arduino UNO generated pulse width modulation (PWM) signals for the MOSFETs (model: YYNMOS-4, manufactured by YEYY Co., Dongguan, China), which supplied voltages to the left and right SMA wires on the bionic joint, respectively. The voltage provided to the MOSFETs was 4.5 V. The Arduino UNO, MOSFETs, and rotary encoder shared a common signal ground. The fan (model: KRD-USB08, power supply 5 V 2.5 W, average wind speed measured by GM816A digital anemometer: 2.9 m/s, manufactured by KRD Co., Hefei, China), operating at a voltage of 5 V and provided by the Arduino UNO, effectively generated forced air convection for the SMA wires of the bionic joint.

### 4.2. Open-Loop Simulation and Experiment

The open-loop simulation model was from Section 3, utilizing the parameters presented in Table 2, which were derived from [26] and actual measurements. Both the open-loop simulation and experiment were conducted with single-sided SMA wires heating at an actuating voltage of 4.5 V, over a total time of 15 s. During this period, the power-on heating time was 2 s, with the remaining time dedicated to power-off cooling.

**Figure 8:**
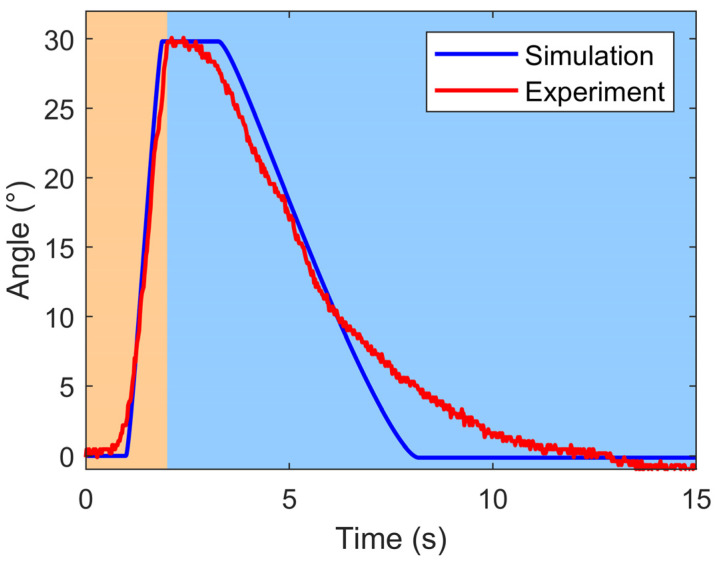
Results of open-loop simulation and experiment for the bionic joint (the light orange area represents the power-on heating phase, while the light blue area indicates the power-off cooling phase).

The results of the open-loop simulation and experiment for the bionic joint are depicted in Figure 8. The simulation outcomes exhibited relative smoothness, whereas the experimental data exhibited some jitteriness. Despite this, both datasets exhibited a consistent overall trend. During the power-on heating stage of the SMA wires, an initial joint angle change exhibited a brief hysteresis segment. As the SMA wires heated up to reach the austenite start temperature of the SMA wire, the joint angle rapidly increased to approximately 30°. In the power-off cooling stage of the SMA wires, there was also a hysteresis segment upon initial joint angle change, followed by a gradual recovery. Subsequently, the joint angle decreased to approximately 0°. This hysteresis phenomenon was attributed to the inherent phase transformation characteristics of the SMA wire, which required some time to reach the initial temperature for phase transformation of the SMA wire.

### 4.3. Position Step Response

The positional step response experiment examined the performance of the bionic joint in response to a specific step input. The step input involved gradually increasing the rotation angle from 0° in increments of 10° every 5 s until it attained 30°. Afterward, the angle was decreased from 30° in decrements of 10° every 5 s until it reached −30°. Ultimately, it was increased from −30° in increments of 10° every 5 s until it returned to 0°. The gains for the experiment were established as $k_{P_{1}}=-k_{P_{2}}=-0.125$ and $k_{I_{1}}=-k_{I_{2}}=-0.0004$. The input voltage $V_{IN_{i}}$ was set to 4.5 V. Additionally, forced air convection was set up for the SMA wires on the bionic joint throughout the experiment.

**Figure 9:**
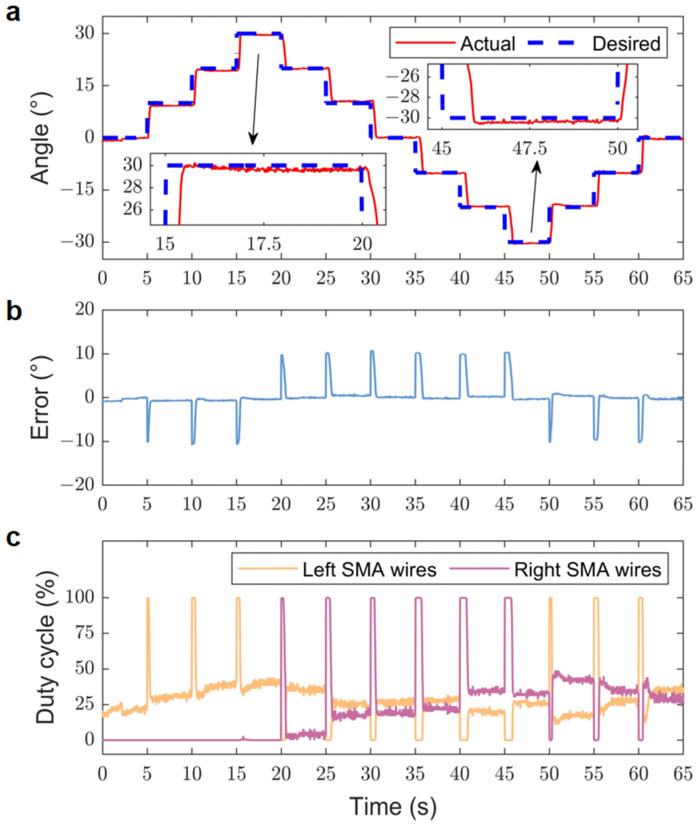
Experimental results of the position step response. (**a**) Actual and desired positions. (**b**) Position error. (**c**) Duty cycles corresponding to the SMA wires.

Figure 9 presents the experimental results of the position step response of the bionic joint. The experimental data reveal that the bionic joint had an average step response time of 0.77 s, with a root mean square error (RMSE) of 2.84°. Specifically, the average response time for the increase process in the first phase was 0.37 s, while the decrease process in the second phase took 0.95 s, and the increase process in the third phase averaged 0.78 s.

Among the three stages, the first stage demonstrated the shortest average response time due to the fact that only one side of the SMA wires was energized and heated. Conversely, the last two stages involved both the left and right SMA wires in stepping and maintaining stability, resulting in longer response times.

By comparing Figure 9a and Figure 9b, it is evident that the time required for each step transition gradually increased across the three aforementioned phases, with an accumulation of error, particularly noticeable in the second phase. The peak error typically occurred during the step response. Additionally, delays and overshoots were also observed.

One potential explanation for these observations is the error accumulation in the PI controller. Furthermore, the inherent hysteresis in the heating and cooling process of the SMA wires could have also contributed to these delays and overshoots.

### 4.4. Position Tracking Response

The positional tracking response experiment aimed to assess the tracking response performance of the bionic joint by having it follow sinusoidal trajectory inputs at varying frequencies. The positional tracking response of the bionic joint reflects its capability to track a predefined trajectory swiftly and precisely. The gains for the experiment were established as $k_{P_{1}}=-k_{P_{2}}=-0.3$ and $k_{I_{1}}=-k_{I_{2}}=-0.1$. The input voltage $V_{IN_{i}}$ was set to 4.5 V. Additionally, throughout the experiment, forced air convection was also established for the SMA wires located on the bionic joint. The sinusoidal trajectory input frequencies utilized in the experiments were 0.05 Hz and 0.1 Hz, with amplitudes set to 30° for both frequencies.

**Figure 10:**
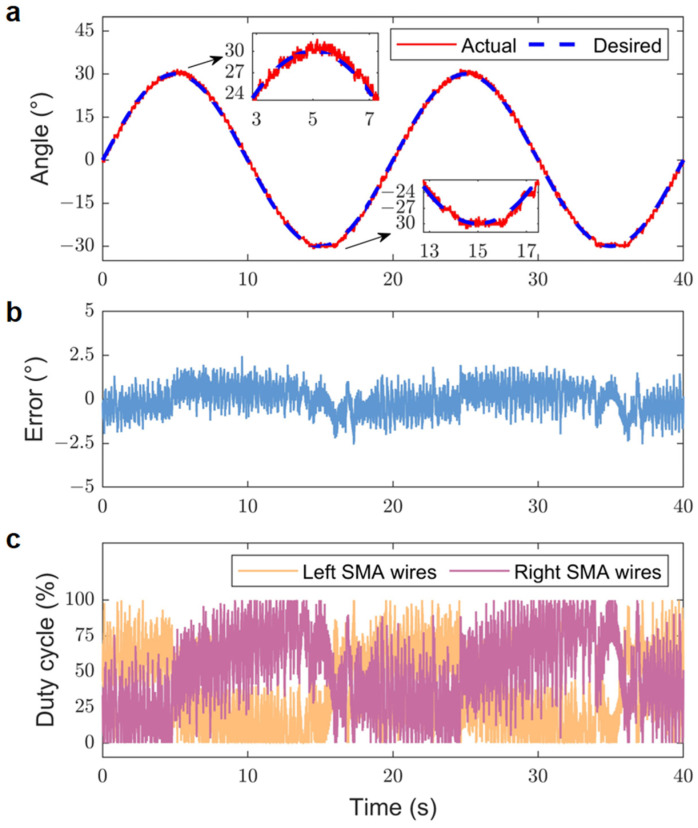
Experimental tracking results for 0.05 Hz sinusoidal desired trajectory. (**a**) Actual and desired positions. (**b**) Position error. (**c**) Duty cycles corresponding to the SMA wires.

Figure 10 illustrates the experimental tracking results for the 0.05 Hz sinusoidal desired trajectory. The bionic joint was able to follow the prescribed trajectory, albeit with some jitter present. The RMSE of the tracking result for this trajectory was 0.77°, and the absolute peak error reached approximately 2.56°. This peak error occurred at the sinusoidal peak and was likely due to the hysteresis effects associated with the heating and cooling of the SMA wires.

**Figure 11:**
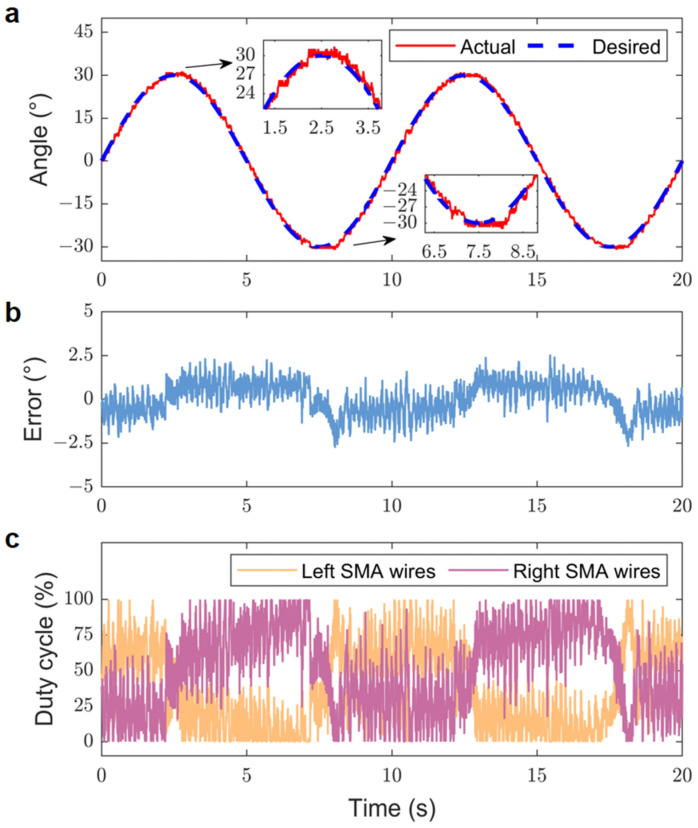
Experimental tracking results for 0.1 Hz sinusoidal desired trajectory. (**a**) Actual and desired positions. (**b**) Position error. (**c**) Duty cycles corresponding to the SMA wires.

Figure 11 displays the experimental tracking results for the 0.1 Hz sinusoidal desired trajectory. In this case, the RMSE of the tracking results increased to 0.93°, and the absolute peak error rose to approximately 2.77°.

A comparison of the tracking results for the bionic joint following sinusoidal desired trajectories at different frequencies, specifically 0.05 Hz and 0.1 Hz, revealed that the tracking error gradually increased with rising frequency. The tracking trajectory of the bionic joint was constrained by a fixed cycle time. Due to the hysteresis of the SMA wires, the bionic joint exhibited a slight lag behind the sinusoidal expected trajectory, particularly at the peak of the sinusoidal movement.

### 4.5. Differential SMA Wire-Actuated Bionic Joint Performance Comparison

Previous studies have also showcased notable designs for differential SMA wire-actuated bionic joints, all demonstrating exceptional design and performance qualities. Table 3 presents a concise summary of several existing bionic joints, highlighting essential parameters such as the pulley radius, effective length of SMA wires, and the maximum rotation angle achieved by the joint design.

As presented in Table 3, a comparison revealed that the bionic joint proposed in this paper effectively utilized the stroke amplification provided by the differential movable pulley set, allowing for the use of shorter SMA wires compared to the other three bionic joints mentioned in the table, while attaining a significant rotation angle. Although our trajectory tracking error is relatively larger, this is primarily due to the larger amplitude of the tracking. Nevertheless, our tracking accuracy of the bionic joint is still quite satisfactory.

## 5. Conclusions

In this work, a bionic joint based on SMA wires and a differential pulley set structure was proposed. A differential pulley set structure, which amplifies the stroke of the SMA wires, was introduced to enhance the rotation angle of the bionic joint. Traditionally, due to the limited strain of SMA wires, long SMA wires were used in bionic joints to achieve a large rotation angle. However, our approach amplifies the stroke of the SMA wires, potentially reducing the length of SMA wires required for the desired motion. Such a design offers promising applications in robotic systems, including small robot joints and dexterous hand designs. A mathematical model of the bionic joint was derived. Utilizing the bionic joint angle as feedback, a PI controller was designed to generate the appropriate voltages for the SMA wires. The results of the open-loop simulation and experiment were compared. Position step response and position tracking response experiments were conducted to assess the control performance of the bionic joint. The experimental results demonstrated that the bionic joint, under the control of the PI controller, exhibited satisfactory performance in terms of position response (within a range of −30° to 30°).

Although an anti-windup method was employed to mitigate integral windup, some error accumulation was still observed in the results. This could be due to various factors such as system nonlinearities, unmodeled dynamics, sensor noise, or actuator limitations. Additionally, hysteresis was also observed in the SMA wires during the heating and cooling process.

However, the primary focus of this study was to assess the bionic joint’s performance in terms of its operating range and response efficiency; thus, hysteresis was not a central concern. In our future work, we will focus on testing and compensating for hysteresis in SMA wires by employing more advanced control strategies. Further analysis and fine-tuning of the controller parameters are needed to improve the system’s performance and reduce error accumulation. Moreover, we intend to integrate the bionic joint into robotic systems, evaluate its practical applications, and explore its potential in real-world scenarios.

## Figures and Tables

**Table 1:** Key dimensions for bionic joint design.

Dimension	Value (Unit)
Length, width, and height of the bionic joint	60 × 60 × 180 mm
Large radius of the double-diameter fixed pulley	13.2 mm
Small radius of the double-diameter fixed pulley	11.2 mm
Radius of the V-groove bearing within each movable pulley	4.2 mm
Length, width, and height of PCBs 1 and 3	10 × 10 × 1.6 mm
Length, width, and height of PCBs 2 and 4	17 × 10 × 1.6 mm
Length of each SMA wire	80 mm
Diameter of each SMA wire	0.31 mm

**Table 2:** Parameters in the model of the SMA wire and the bionic joint.

Description (Parameters)	Value (Unit)
Austenite Young’s modulus ($E_{A}$)	78 GPa
Martensite Young’s modulus ($E_{M}$)	23 GPa
Maximum recoverable strain of the SMA wire ($\varepsilon_{L}$)	0.02
Number of parallel SMA wires on each side (n)	2
Diameter of the SMA wire (d)	0.31 mm
Austenite start temperature ($A_{s}$)	80 °C
Austenite finish temperature ($A_{f}$)	98 °C
Martensite start temperature ($M_{s}$)	78 °C
Martensite finish temperature ($M_{f}$)	60 °C
Austenite stress influence coefficients ($C_{A}$)	15 MPa/°C
Martensite stress influence coefficient ($C_{M}$)	15 MPa/°C
Initial martensite volume fraction of the austenite-to-martensite phase transformation ($\xi_{A}$)	0
Initial martensite volume fraction of the martensite-to-austenite phase transformation ($\xi_{M}$)	1
Length of each SMA wire (l)	80 mm
Density of the SMA wire (ρ)	6450 kg/m^3^
Specific heat capacity of the SMA wire ($c_{p}$)	837 J/(kg·°C)
Resistance per unit length of the SMA wire ($R_{0}$)	12.2 Ω/m
Heat convection constant coefficient ($h_{0}$)	80
Heat convection second order coefficient ($h_{2}$)	0.001
Ambient temperature ($T_{0}$)	20 °C
Radius of the large groove on the double-diameter fixed pulley ($r_{1}$)	13.2 mm
Radius of the small groove on the double-diameter fixed pulley ($r_{2}$)	11.2 mm
Moment of inertia of the double-diameter fixed pulley ($J_{1}$)	1.52 kg·(mm)^2^
Mass of the movable pulley ($m_{1}$)	2.56 g
Damping coefficients of SMA wires ($b_{1}$$,\;b_{2}$)	2

**Table 3:** Parameter performance of differential SMA wire-actuated bionic joints.

Model	Pulley Radius	Effective Length of SMA Wires	Maximum Rotation Angle	RMSE in Track Response
Jia [19]	25 mm	380 mm	62◦	-
Guo [29]	15 mm	370 mm	30°	0.5°
Britz [30]	15 mm	196 mm	30°	-
This paper	13.2 mm	80 mm	60°	0.8°

## Data Availability

Data are contained within this article.
